# Cardioprotective Effect of Betulinic Acid on Myocardial Ischemia Reperfusion Injury in Rats

**DOI:** 10.1155/2014/573745

**Published:** 2014-05-21

**Authors:** Anzhou Xia, Zhi Xue, Yong Li, Wei Wang, Jieyun Xia, Tiantian Wei, Jing Cao, Weidong Zhou

**Affiliations:** ^1^Department of Pharmacology, Xuzhou Medical College, Xuzhou, Jiangsu 221004, China; ^2^Department of Gastrointestinal Surgery, Xuzhou Central Hospital, Xuzhou, Jiangsu 221006, China; ^3^Department of Cardiology, The People's Hospital of Suining, Suining, Xuzhou, Jiangsu 221200, China

## Abstract

*Objectives*. This study aims to investigate the effect of betulinic acid (BA) on myocardial ischemia reperfusion/injury in an open-chest anesthetized rat model. *Methods*. The model was induced by 30 minutes left anterior descending occlusion followed by 2 hours reperfusion. There are six groups in our present study: sham operation group, ischemia/reperfusion group, low-dosage BA group, medium-dosage BA group, high-dosage BA group, and fosinopril sodium group. Rats in the latter four groups were administrated with BA (50, 100, and 200 mg/kg, i.g.) or fosinopril sodium (10 mg/kg, i.g.) once a day for 7 days before operation, respectively. Rats in the former two groups were given the same volume of vehicle (0.5% CMC-Na, i.g.). During the operation, cardiac function was continuously monitored. Serum LDH and CK were measured with colorimetric assays. The expression of Bcl-2 and Bax and the apoptosis of cardiomyocytes were investigated with western blot and TUNEL assay, respectively. *Results*. Pretreatment with BA improved cardiac function and attenuated LDH and CK activities compared with IR group. Further investigation demonstrated that the expression of Bcl-2 and Bax and TUNEL assay was in line with the above results. *Conclusion*. BA may reduce the release of LDH and CK, prevent cardiomyocytes apoptosis, and eventually alleviate the extent of the myocardial ischemia/reperfusion injury.

## 1. Introduction

In China, cardiovascular diseases (CVDs) are the leading cause of death and a major health problem. According to the report on cardiovascular diseases in China, approximately 3 million Chinese people die from CVD every year, accounting for 40% of all causes of death [1], of which ischemic heart disease accounts for a large percentage. Ischemic heart disease is a major cause of morbidity and mortality in both the developing and the developed world now [2]. Myocardial ischemia/reperfusion injury (MIRI) was first postulated in 1960 by Jennings et al. [3], which refers to a phenomenon that timely restoring coronary blood flow after myocardial ischemia induces severe myocardium injury, although at the same time it could reduce myocardial infarct size and improve the clinical outcomes. The underlying mechanisms are complex, and the present main proposals include Ca^2+^ overload, excessive reactive oxygen species (ROS) generation, inflammation, and apoptosis [4]. These modalities interact with each other, and apoptosis plays a pivotal role in the progress of MIRI and, thus, influences the outcomes. Apoptosis is usually triggered by intracellular Ca^2+^ overload, which induces the processing of procaspase-8 into active caspase-8 and the activation of Bax, which lead to the release of the apoptosis-inducing factor, Smac, and cytochrome-c from mitochondria. Apoptosis-inducing factor translocates into the nucleus and facilitates nonspecific DNA fragmentation. Smac inactivates X chromosome-linked inhibitor of apoptosis protein, which inhibits caspase-3, and cytochrome-c forms an apoptosome complex with procaspase-9 and apoptotic protease-activating factor-1, which activates caspase-9. Taken together, these cascades ultimately contribute to irreversible cellular dysfunction [4].

As a result of this paradoxical phenomenon, studies in this area focus on how to eliminate or at least partly diminish the injury, and, fortunately, ischemic preconditioning and ischemic postconditioning were documented as effective strategies to reduce MIRI. Both of these pathways, however, have ethical questions. Recently, lots of pharmacological agents, such as adenosine, bradykinin, opioids, glucagon-like peptide 1, atrial natriuretic peptide, insulin, volatile anesthetics, nitroglycerin atorvastatin, nicorandil, and ciclosporin, have also been verified that they could protect myocardium from MIRI [2], and it is termed as pharmacological preconditioning/postconditioning.

Betulinic acid, serving as a pentacyclic triterpene, has several botanical sources, and it can also be derived chemically from betulin, a substance found in abundance in the outer bark of white birch trees (*Betula alba*) [5, 6]. The compound is mainly known for its antitumor [7–9] and anti-inflammatory [10, 11] activities. Recent studies have shown that BA protects against cerebral [12] and renal [6] ischemia reperfusion injuries. However, effect of BA on myocardial ischemia reperfusion injury has not been demonstrated yet. Thus, the objectives of our study were (1) whether BA protects against myocardial ischemia/reperfusion and (2) if BA actually has cardioprotective activity, what are the underlying mechanisms?

## 2. Materials and Methods 

### 2.1. Animals and Reagents

Male Sprague-Dawley (SD) rats (Laboratory Animal Centre, Xuzhou Medical College, Xuzhou, China), weighing 220–240 g, were used for these experiments. The animals were housed on a 12 h light/dark cycle under controlled temperature (23 ± 1°C) and relative humidity (65–70%). The animals were randomly divided into specified experimental groups. The procedures in this study were conducted in accordance with the Chinese Council on Animal Care and Institutional Care Committee of Xuzhou Medical College.

BA (purity > 98%) was purchased from Nanjing Spring & Autumn Biological Engineering Co., Ltd. (Jiangsu, China). Fos was purchased from China and American Shanghai Squibb Company (Shanghai, China). All other reagents were of standard analytical grade.

### 2.2. Experimental Protocols

Forty-eight SD rats were randomly divided into six groups as follows: (1) Sham operation group (Sham, *n* = 8); (2) IR group (heart subjected to ischemia/reperfusion, *n* = 8); (3) BA L group (heart subjected to ischemia/reperfusion treated with low-dose betulinic acid, 50 mg/kg/d, *n* = 8); (4) BA M group (heart subjected to ischemia/reperfusion treated with medium-dose betulinic acid, 100 mg/kg/d, *n* = 8); (5) BA H group (heart subjected to ischemia/reperfusion treated with high-dose betulinic acid, 200 mg/kg/d, *n* = 8); (6) Fos group (heart subjected to ischemia/reperfusion treated with fosinopril sodium, 10 mg/kg/d, *n* = 8). Rats in latter four groups were administrated with drug (once a day, i.g., 1 mL/100 g) for 7 days before the operation. And rats in the Sham group and IR group were given equal volumes of 0.5% CMC-Na at the same time. Prior to surgical procedure, rats were anesthetized by intraperitoneal injection of 10% chloral hydrate (350 mg/kg). IR model was induced by ligating the left anterior descending (LAD) for 30 min followed by reperfusion for 2 h in anesthetized rats. The significant fall of the ST segment of the ECG was selected as the reperfusion criterion. Rats in the Sham group underwent the same surgical procedures except that the suture placed under the LAD was not tied.

### 2.3. Hemodynamic Measurement

Cardiac function was continuously monitored before and during the entire IR procedure by PowerLab 16/30 data acquisition system (AD Instrument, Germany). And the data at baseline, 30 min of ischemia, and 30, 60, 90, and 120 min of reperfusion were analyzed. Left ventricular systolic pressure (LVSP), left ventricular end-diastolic pressure (LVEDP), heart rate, and the rate in rise and fall of ventricular pressure (±*dp*/*dt*_max⁡_) were recorded as left ventricular functional parameters.

### 2.4. Measurement of Serum LDH and CK Activities

Blood was collected from abdominal aorta immediately after reperfusion for 2 hours. Lactate dehydrogenase (LDH) and creatine kinase (CK) assay kits (Jiancheng Bioengineering Institute, Nanjing, China) were used to detect their activities. All procedures were according to the manufacturer's protocol.

### 2.5. Histologic Examination

The heart was fixed in 10% neutral-buffered formalin, embedded in paraffin, and cut into 4 *μ*m sections. The sections were stained using hematoxylin and eosin (H&E) for histochemistry. All histopathological changes were evaluated in a blinded fashion by two investigators, and the main observation indexes including intercellular space, edema of cardiomyocytes, and inflammatory cell infiltration were assessed under microscope (OLYMPUS, Japan).

### 2.6. Assessment of Apoptosis

Apoptosis was detected by the terminal deoxyribonucleotide transferase- (TdT-) mediated dUTP nick end labeling (TUNEL) detection kit (Roche, Germany) according to the manufacturer's protocol. In this method, the TUNEL-positive brown-colored cells were considered to be apoptotic cells. The results were scored semiquantitatively by averaging the number of apoptotic cells/field at 400x magnification. Five fields were evaluated per tissue sample, and the cardiomyocytes apoptosis was represented as apoptosis index (AI) calculated as follows: AI = the number of TUNEL-positive cells/the total number of cells.

### 2.7. Western Blot Assay for Bcl-2 and Bax Expression

The samples were ground with RIPA buffer and the mixture was centrifuged at 12,000 g for 15 min at 4°C. The supernatants were stored at −20°C and the protein concentration was measured using bicinchoninic acid (BCA) protein assay kit (Beyotime Institute of Biotechnology). Totally, 100 *μ*g of proteins was separated using 12% sodium dodecyl sulfate polyacrylamide gel electrophoresis (SDS-PAGE). The proteins were transferred to nitrocellulose membranes. The membranes were treated with blocking buffer (3% BSA) for 2 h and incubated with the primary antibodies against Bcl-2 (1 : 100, Beijing Golden Bridge Biotechnology Company, China), Bax (1 : 100, Beijing Golden Bridge Biotechnology Company, China), or GAPDH (1 : 6000, Bioworld, China) at 4°C overnight, respectively. After five times of washing for 5 min with washing buffer, the membranes were incubated with anti-rabbit IgG (Beijing Golden Bridge Biotechnology Company, China) at a ratio of 1 : 1000 at room temperature for 2 h. After another five times of washing for 5 min with washing buffer the membranes were shown by NBT/BCIP. The protein bands were scanned and quantified using Image J software.

### 2.8. Statistical Analysis

Data were presented as means ± SD; statistical analysis was performed with SPSS 13.0. Statistical significance (*P* < 0.05) for each variable was estimated by Student's unpaired *t*-test or one-way analysis of variance (ANOVA) followed by a Bonferroni post hoc correction between all groups.

## 3. Results 

### 3.1. Effects of Betulinic Acid on Left Ventricular Function

**Figure 1:**
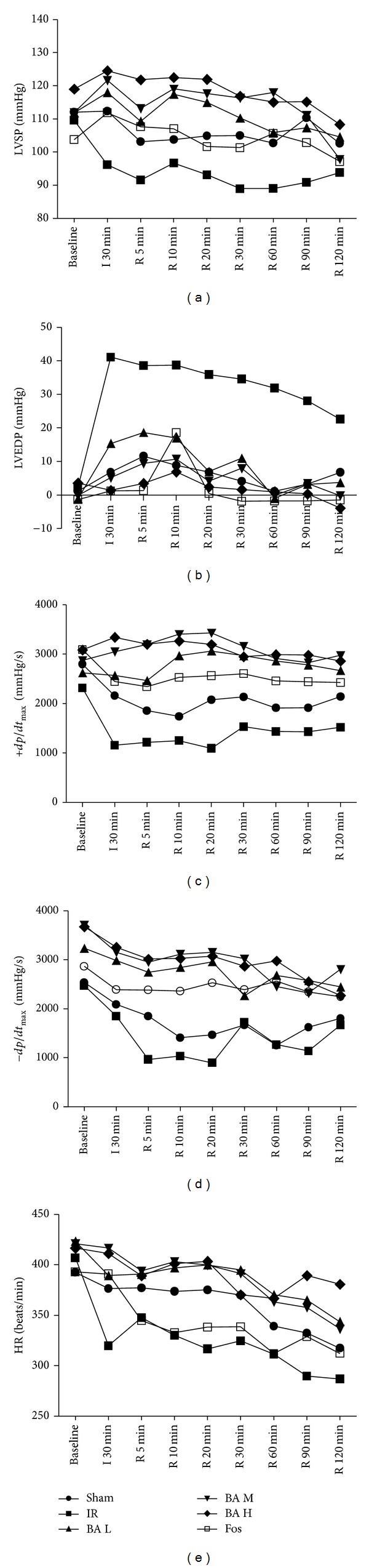
Hemodynamic parameters during the experiments (mean, *n* = 8). LVSP: left ventricular systolic pressure; LVEDP: left ventricular end-diastolic pressure; ±*dp*/*dt*_max⁡_: the rate in rise and fall of ventricular pressure; HR: heart rate.

The hemodynamic data including left ventricular systolic pressure (LVSP), left ventricular end-diastolic pressure (LVEDP), the rate in rise and fall of ventricular pressure ±*dp*/*dt*_max⁡_), and heart rate (HR) were summarized in Table 1 and Figure 1. During ischemia/reperfusion, IR group rats showed lower LVSP than Sham group at R 90 min (Table 1, *P* < 0.01) and BA M group showed increased LVSP at R 30 min and R 60 min (Table 1, *P* < 0.05 versus IR group); however, BA H group increased HR in the phase of I 30 min to R 60 min (Table 1, *P* < 0.05 versus IR group). The LVEDP of IR group was higher than that of Sham group throughout the experimental period (Table 1, *P* < 0.001); interestingly, other groups decreased LVEDP pronouncedly compared with IR group at the same time points (Table 1, *P* < 0.001). +*dp*/*dt*_max⁡_ of rats in IR group was lower than that of Sham group at I 30 min (Table 1, *P* < 0.05). BA groups exert more robust protection against augmentation of +*dp*/*dt*_max⁡_ than Fos group when compared with IR group, but there are no pronouncedly differences between three BA groups. −*dp*/*dt*_max⁡_ of rats in IR group was lower than that of Sham group (Table 1, *P* > 0.05). −*dp*/*dt*_max⁡_ of rats in BA M and BA H groups was higher than that of IR group during almost all the experimental periods (Table 1, *P* < 0.05 or *P* < 0.01). Throughout ischemia/reperfusion experimental period, HR was not significantly different between Sham and IR groups (Table 1, *P* > 0.05). However, BA and Fos increased HR when compared with IR group. BA played strong roles at both ischemia and reperfusion phases, whereas Fos played a role mainly at the end of ischemia (Table 1, *P* < 0.05). Most importantly, at the end of reperfusion, only BA H group markedly increased HR compared with IR group (Table 1, *P* < 0.01).

### 3.2. Effects of Betulinic Acid on LDH and CK Activities

**Figure 2:**
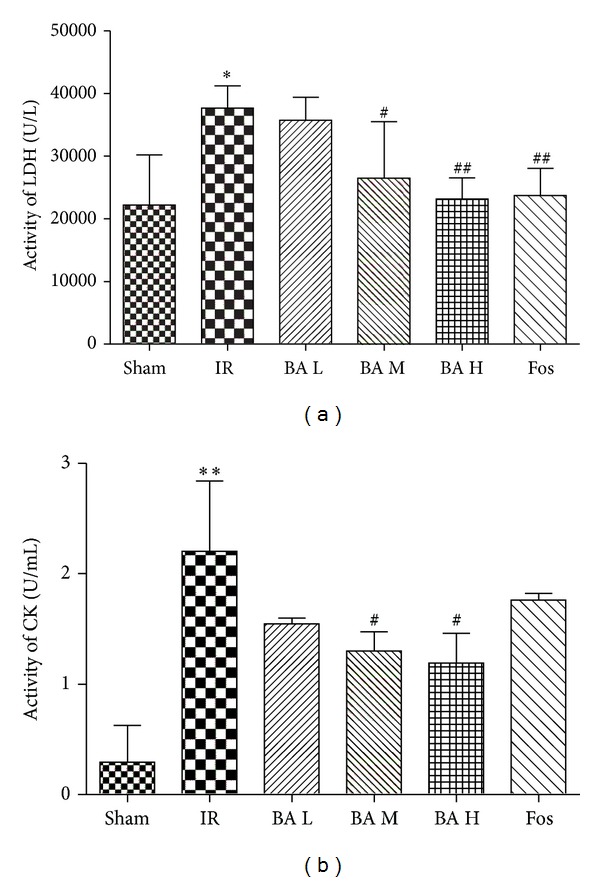
Activities of serum LDH (a) and CK (b) in different groups (mean ± SD, *n* = 8). LDH: lactate dehydrogenase; CK: creatine kinase. *P < 0.05, **P < 0.01 versus Sham group; ^#^ *P* < 0.05, ^##^ *P* < 0.01 versus IR group.

LDH and CK had low activities in Sham group rats serum, but rats subjected to IR injury showed 1.7-fold LDH activities (Figure 2(a), *P* < 0.05) and 7.5-fold CK activities (Figure 2(b), *P* < 0.01) higher than Sham group. In contrast, BA M and BA H groups of rats exhibited decreased both LDH activities (Figure 2(a), *P* < 0.05 or *P* < 0.01) and CK activities (Figure 2(b), *P* < 0.05) compared with IR group. Especially, BA H (200 mg/kg) group decreased LDH and CK to 1.04-fold and 4.06-fold higher than Sham group, respectively.

### 3.3. Effects of Betulinic Acid on Histology

**Figure 3:**
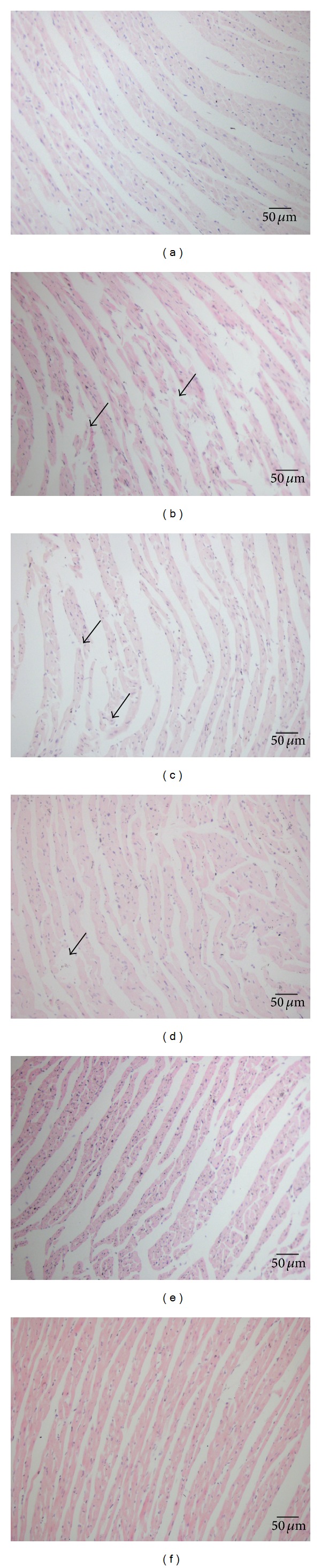
HE staining (×200). (a) Sham group, (b) IR group, (c) BA L group, (d) BA M group, (e) BA H group, and (f) Fos group. The black arrows represent the inflammatory cells.

Rats in the Sham group showed normal architecture of myocardium, cardiomyocytes presented normal size, clear boundaries and arranged regularly, whereas, in IR group, cardiomyocytes arranged irregularly, presented extensive edema, intercellular space enlarged, and inflammatory cell infiltration increased. The changes of the myocardium in the BA-treated and Fos-treated groups were significantly relieved compared with those of the IR group, cardiomyocytes ranked in order, presented mild edema and inflammatory cell infiltration reduced markedly (Figure 3).

### 3.4. Effects of Betulinic Acid on Cardiomyocyte Apoptosis

**Figure 4:**
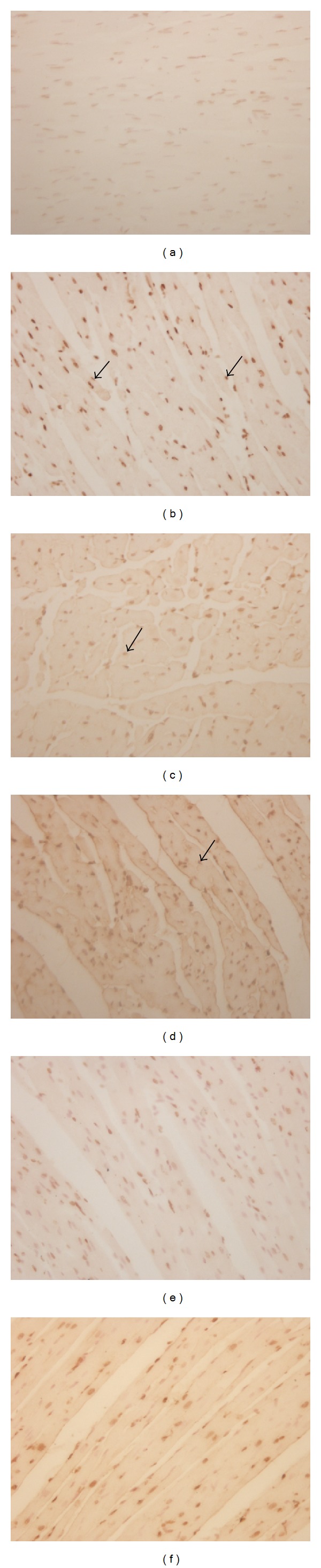
Representative photomicrographs of TUNEL assay (×400). (a) Sham group, (b) IR group, (c) BA L group, (d) BA M group, (e) BA H group, and (f) Fos group. The black arrows represent positive apoptotic cells.

**Figure 5:**
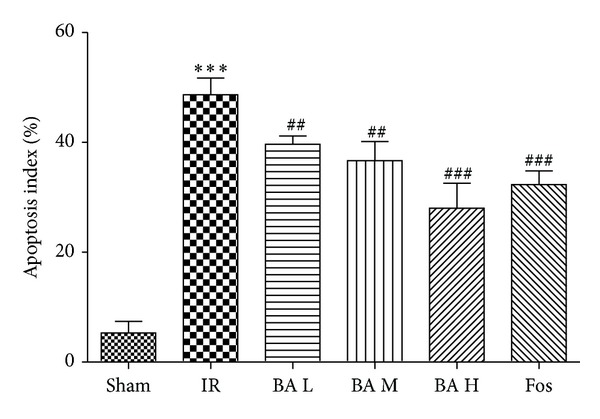
Apoptosis index of the six groups (mean ± SD, *n* = 8). ***P < 0.001 versus Sham group; ^##^ *P* < 0.01, ^###^ *P* < 0.001 versus IR group. Apoptosis index (AI) = the number of positively stained apoptotic myocytes/the total number of myocytes counted × 100%.

The presence of apoptotic cells was documented by the TUNEL assay (Figure 4). In Sham group, only scattered TUNEL-positive cells were observed. The hearts of animals that were subjected to myocardial ischemia reperfusion exhibited severe tissue damage appeared to have increased number of TUNEL-positive cells (Figure 5, 48.67 ± 4.27% versus 5.33 ± 1.37%, *P* < 0.001). In contrast, BA and Fos groups of rats demonstrated a marked reduction of TUNEL-positive cells compared with IR group (Figure 5, BA L: 39.83 ± 3.97%, *P* < 0.01; BA M: 38.50 ± 3.73%, *P* < 0.01; BA H: 27.83 ± 4.45%, *P* < 0.001; Fos: 32.00 ± 5.37%, *P* < 0.001).

### 3.5. Effects of Betulinic Acid on the Expression of Bcl-2 and Bax

**Figure 6:**
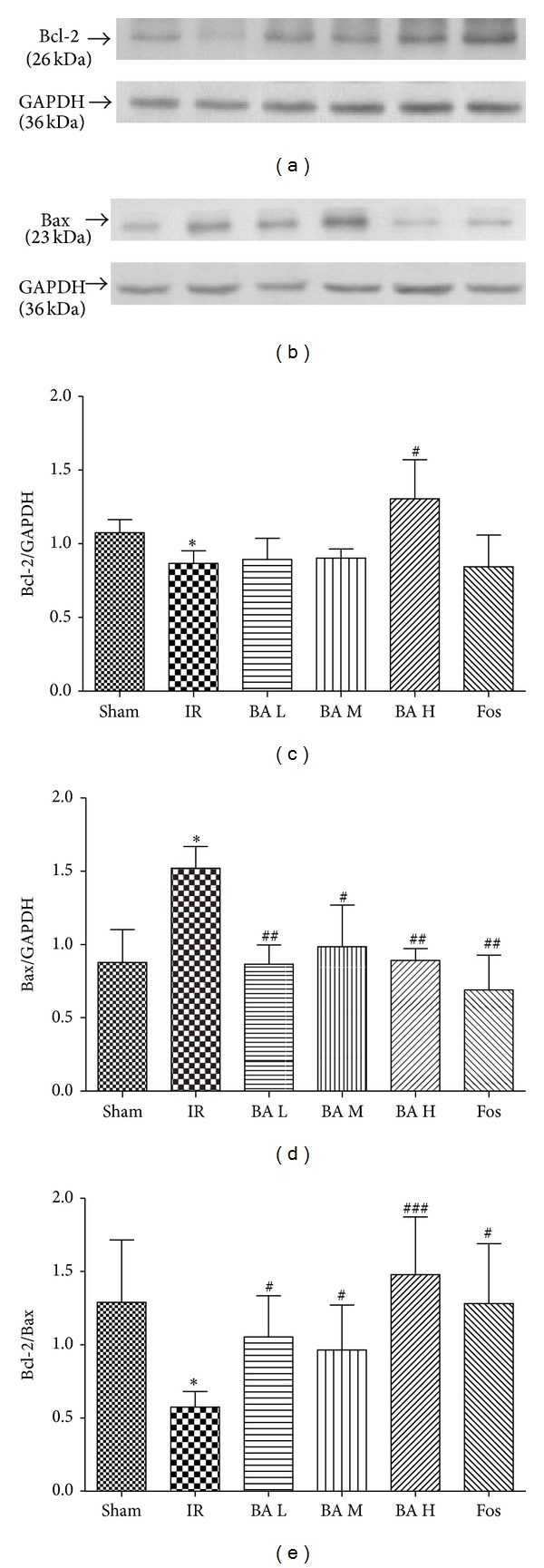
Expression of Bcl-2 (a) and Bax (b) by Western blot analysis. (c) Quantification of the Bcl-2/GAPDH. (d) Quantification of the Bax/GAPDH. (e) Quantification of the Bcl-2/Bax (mean ± SD, *n* = 3). *P < 0.05 versus Sham group; ^#^ *P* < 0.05, ^##^ *P* < 0.01, ^###^ *P* < 0.001 versus IR group.

Sham group demonstrated basic expression of both Bcl-2 and Bax and IR induced downregulated Bcl-2 and upregulated Bax (Figures 6(c) and 6(d), *P* < 0.05). Pretreating with BA (especially high dosage) increased the expression of Bcl-2 (Figure 6(c), *P* < 0.05) and decreased the expression of Bax (Figure 6(d), *P* < 0.01) and, consequently, upregulated Bcl-2/Bax ratio compared with IR group (Figure 6(e), *P* < 0.001).

## 4. Discussion

It has been verified that BA possesses antitumor and anti-inflammatory activities and recently cerebral and renal ischemia reperfusion injuries protection. However, effects of BA on myocardial ischemia reperfusion injury have not been elucidated clearly yet. In the present study, we showed the* in vivo* evidence for the first time that BA reduces the release of LDH and CK, suppresses myocardial apoptosis, alleviates ischemia/reperfusion injury, and therefore improves left ventricular function. These results suggest that pretreatment with BA may play an important role in reducing myocardial ischemia/reperfusion injury in rats.

Ischemic preconditioning was documented as effective strategies to reduce MIRI, but this pathway has ethical questions. Preconditioning has two protection stages: early stage which starts in 30 min to several hours and late stage which lasts between 2 and 3 days. For the early stage, it is caused by immediate signal transduction through opioid receptor [13], bradykinin [14], and adenosine receptor [15]. However, the late stage is induced by transcription and translation. Previous studies indicate that BA pretreatment could alleviate the myocardial injury, suppress cardiomyocyte apoptosis, and improve cardiac function; obviously it could induce the early stage protection against MIRI. It was suggested that the late stage protection of preconditioning is associated with the activity of endogenous antioxidant enzymes, such as SOD, catalase, and the level of HSP 70. BA has significantly antioxidant activity which could reduce the consumption of endogenous antioxidant enzymes, so it also could induce the late stage protection of preconditioning. Moreover antiapoptotic properties of BA pretreatment also contribute to the late stage protection against ischemia/reperfusion insult.

Left ventricular remodeling which starts immediately after acute myocardial infarction and evolves in the chronic phase of heart failure associates with decrease in cardiac function and ventricular dilation [16]. Impairment of ventricular function is the most common fatal complication secondary to ischemic heart diseases. Therefore, improvement of cardiac function and attenuation of ventricular dilation are vital for the treatment of ischemic heart diseases [17]. The present study demonstrated that BA, especially the high dose of BA (200 mg/kg), could increase LVSP, HR, and ±*dp*/*dt*_max⁡_ but decrease LVEDP (Table 1 and Figure 1). It is known that LVSP and +*dp*/*dt*_max⁡_ represent ventricular systolic function and LVEDP and −*dp*/*dt*_max⁡_ refer to ventricular diastolic function. We indicated that BA could improve recovery of left ventricular function from improving both systolic and diastolic functions.

It has been shown that LDH and CK are reliable markers of cellular necrosis [18–20] and CK was also well known to be a golden criterion of cardiomyocyte injury; CK measurement plays a vital role in early diagnosis of myocardial infarction and other diseases, and it is more reliable than ECG analysis. In our study, determining two kinases associated with cardiomyocytes injury has two purposes: (1) to make sure successful establishment of IR model and (2) wonder if BA exerts its cardioprotective effect through attenuating the LDH and CK activities. Our results were in accordance with the two objectives mentioned above (Figure 2).

Myocardial ischemia and reperfusion injury leads to cell death [21]. And accumulated evidence indicated that apoptosis, a complex series of ordered cell-autonomous biochemical events, contributes significantly to myocardial cell death, suggesting that therapeutic interventions that inhibit apoptotic cell death may attenuate ischemic-induced heart injury [16, 22]. The Bcl-2 family consists of pro- and antiapoptotic members. The family consists of both cell death promoters such as Bax and Bad and cell death inhibitors, which include Bcl-2 and Bcl-X. It has been demonstrated that the high ratio of Bax/Bcl-2 is associated with greater vulnerability to apoptotic activation [17, 23–26]. The balance between proapoptotic and antiapoptotic proteins determines the possibility of cells to either survive or undergo apoptosis after a certain stimulus or injury [27]. Since inhibition of the apoptotic processes has been shown to prevent the myocardial ischemia/reperfusion injury, we next studied the effect of BA on apoptosis through TUNEL assay (Figures 4 and 5). As shown in Figure 4, we found that BA could inhibit myocardial cells apoptosis, expressed as decreased number of TUNEL-positive cardiomyocytes. Furthermore, we suggested that the myocardial apoptosis which we examined is early apoptosis and it can initiate extensive loss of cardiomyocytes, contribute to the pathogenesis of MIRI, and ultimately deteriorate cardiac function. Meanwhile, the expressions of Bax and Bcl-2 were measured by Western blot (Figure 6), and the results show that BA may upregulate the expression of Bcl-2 while downregulating Bax expression, therefore increasing the ratio of Bcl-2 to Bax (Figure 6). Interestingly, treatment with 200 mg/kg of BA significantly prevents myocardial cells undergoing apoptosis after ischemia/reperfusion insult in this study based on statistical analysis.

Our study has some limitations. Firstly, mechanisms underlying the cardioprotective effect of BA need further investigations. It has been reported that BA could suppress oxidant stress. Here, we only focus on Bcl-2/Bax ratio, a classical pathway in apoptosis research, but if there are other signaling pathways involved in the cardioprotection of BA? We do not know whether it has reached to your magazine's standard. Secondly, we did not show infarct size (IS) measurement results. IS measurement is a common and direct item to weigh cardioprotective effect, but rats in our present study were limited; we plan to measure it in our further researches.

Based on the above results, we suggested that BA ameliorates myocardial ischemia/reperfusion injury in rats by inhibiting the release of LDH and CK, suppressing myocyte apoptosis. This may provide insight into the role of BA in MIRI.

## Figures and Tables

**Table 1:** Hemodynamic parameters during the experiments ($\overset{-}{x}\pm s,n=8$).

Parameters	Baseline	I 30 min	R 30 min	R 60 min	R 90 min	R 120 min
LVSP (mmHg)						
Sham	112.04 ± 4.14	112.38 ± 6.94	105.00 ± 10.49	102.73 ± 11.59	110.35 ± 6.59	102.69 ± 15.56
IR	109.60 ± 8.79	96.16 ± 8.26	88.96 ± 5.55	89.00 ± 7.92	90.88 ± 9.97**	93.84 ± 4.41
BA L	111.83 ± 16.32	117.98 ± 17.12	110.28 ± 20.84	105.87 ± 26.25	107.31 ± 16.88	104.59 ± 18.16
BA M	111.94 ± 12.05	121.68 ± 5.66	116.31 ± 16.24^#^	118.04 ± 18.63^#^	111.18 ± 21.05	97.89 ± 10.58
BA H	119.00 ± 9.99	124.58 ± 9.48^#^	116.89 ± 16.53^#^	115.06 ± 11.78^#^	115.18 ± 18.72	108.35 ± 21.23
Fos	103.75 ± 18.86	111.85 ± 6.00	101.37 ± 22.47	105.61 ± 16.36	102.85 ± 20.80	97.17 ± 20.11
LVEDP (mmHg)						
Sham	1.17 ± 5.06	6.83 ± 1.88	4.10 ± 3.69	1.13 ± 2.92	3.29 ± 6.03	6.76 ± 5.43
IR	2.40 ± 0.67	41.09 ± 5.01***	34.56 ± 4.41***	31.89 ± 3.52***	28.08 ± 3.02***	22.65 ± 2.47***
BA L	−1.10 ± 1.88	15.35 ± 4.29^###^	10.99 ± 2.73^###^	−0.97 ± 1.30^###^	3.22 ± 1.51^###^	3.73 ± 1.08^###^
BA M	−0.12 ± 0.88	5.18 ± 0.99^###^	7.95 ± 4.73^###^	−0.14 ± 1.54^###^	3.52 ± 0.82^###^	−0.17 ± 0.55^###^
BA H	3.57 ± 0.79	1.44 ± 0.38^###^	1.68 ± 0.63^###^	0.96 ± 0.29^###^	0.39 ± 1.84^###^	−3.85 ± 4.78^###^
Fos	−1.36 ± 3.46	1.22 ± 4.72^###^	−1.89 ± 1.20^###^	−1.79 ± 0.74^###^	−1.78 ± 1.81^###^	−1.49 ± 2.36^###^
+*dp*/*dt*_max⁡_ (mmHg/s)						
Sham	2796.99 ± 747.48	2159.27 ± 616.19	2133.73 ± 664.39	1913.15 ± 746.26	1914.93 ± 740.10	2140.04 ± 904.89
IR	2314.22 ± 523.11	1158.08 ± 126.28*	1530.25 ± 457.50	1436.53 ± 335.40	1431.64 ± 396.76	1516.97 ± 301.25
BA L	2622.34 ± 554.96	2567.47 ± 783.53^##^	2966.97 ± 727.40^##^	2862.31 ± 929.28^##^	2780.30 ± 676.20^##^	2669.20 ± 607.90^#^
BA M	2872.46 ± 900.11	3050.93 ± 530.30^###^	3163.21 ± 549.83^###^	2912.10 ± 761.54^##^	2827.94 ± 631.95^##^	2974.98 ± 607.48^##^
BA H	3084.91 ± 652.90	3340.11 ± 416.84^###^	2952.91 ± 562.36^##^	2992.94 ± 266.80^###^	2982.98 ± 418.88^###^	2860.16 ± 626.23^##^
Fos	2671.93 ± 811.72	2445.77 ± 495.77^##^	2604.37 ± 1088.58^#^	2459.49 ± 906.66	2440.16 ± 593.09	2425.22 ± 520.44
−*dp*/*dt*_max⁡_ (mmHg/s)						
Sham	2538.50 ± 842.98	2088.54 ± 804.64	1667.45 ± 538.83	1255.24 ± 521.60	1621.34 ± 727.98	1804.14 ± 766.69
IR	2470.82 ± 710.12	1851.84 ± 511.17	1724.73 ± 154.39	1263.52 ± 202.00	1137.58 ± 215.10	1670.25 ± 353.45
BA L	3235.65 ± 907.33	2985.13 ± 649.47^##^	2264.23 ± 654.76	2681.89 ± 918.39^###^	2574.89 ± 615.74^###^	2444.46 ± 570.20
BA M	3716.18 ± 615.38^##^	3155.59 ± 305.19^###^	3020.08 ± 527.96^##^	2455.59 ± 427.79^##^	2321.44 ± 448.27^##^	2809.39 ± 734.00^##^
BA H	3673.42 ± 396.67^##^	3258.30 ± 416.35^###^	2869.15 ± 466.16^##^	2978.49 ± 297.56^###^	2550.87 ± 756.55^###^	2270.48 ± 612.49
Fos	2864.85 ± 610.09	2390.76 ± 677.84	2391.93 ± 718.98	2564.15 ± 514.71^##^	2346.04 ± 577.70^##^	2247.83 ± 513.18
HR (beats/min)						
Sham	392.58 ± 66.04	376.55 ± 35.80	370.02 ± 44.77	339.31 ± 42.13	332.37 ± 56.53	317.47 ± 72.61
IR	406.84 ± 30.06	319.83 ± 70.13	324.68 ± 32.55	311.47 ± 38.66	289.73 ± 58.39	286.94 ± 49.31
BA L	423.22 ± 33.79	389.34 ± 26.38^#^	394.86 ± 15.99^#^	370.48 ± 35.85	365.28 ± 26.74^#^	343.66 ± 34.22
BA M	421.08 ± 34.06	416.78 ± 15.23^###^	391.48 ± 25.79	363.22 ± 26.80	357.79 ± 39.67	336.57 ± 42.96
BA H	416.65 ± 35.79	411.22 ± 29.49^##^	370.43 ± 25.76	366.98 ± 35.70	389.45 ± 50.12^###^	380.80 ± 54.36^##^
Fos	393.02 ± 57.07	390.88 ± 40.24^#^	338.62 ± 59.94	312.03 ± 67.08	328.87 ± 35.22	312.22 ± 57.54

LVSP: left ventricular systolic pressure; LVEDP: left ventricular end-diastolic pressure; ±*dp*/*dt*_max⁡_: the rate in rise and fall of ventricular pressure; HR: heart rate. I: ischemia; R: reperfusion. All data were expressed as the mean ± SD. *P < 0.05, **P < 0.01, ***P < 0.001 versus Sham; ^#^ *P* < 0.05, ^##^ *P* < 0.01, ^###^ *P* < 0.001 versus IR.
